# Investigating the Cognitive Neural Mechanisms of Socially Shared Retrieval-Induced Forgetting: Insights from Behavioral and Electrophysiological Evidence

**DOI:** 10.3390/brainsci16080814

**Published:** 2026-07-31

**Authors:** Xiuqi Chen, Xiquan Qin, Bufan Xu, Yuran Xin

**Affiliations:** Faculty of Psychology, Tianjin Normal University, Tianjin 300387, China; 2330340039@stu.tjnu.edu.cn (X.C.);

**Keywords:** socially shared retrieval-induced forgetting (SS-RIF), retrieval-induced forgetting (RIF), social retrieval practice, inhibitory mechanisms, ERP

## Abstract

**Highlights:**

**What are the main findings?**

- Cognitive activity patterns consistent with the preferred account of cognitive suppression were observed in socially shared retrieval-induced forgetting (SS-RIF).
- SS-RIF exhibited distinct cognitive activity patterns from RIF, as reflected by the absence of significant P2 and FN400 differences between retrieval practice and social interaction phases.

**What are the implications of the main findings?**

- These findings provide cognitive neural evidence consistent with the role of potentially adaptive suppression frameworks in socially shared retrieval-induced forgetting.
- The results indicate that social interaction may shape the temporal characteristics of inhibition, distinguishing SS-RIF from individual retrieval-induced forgetting.

**Abstract:**

**Background:** Social cognition research increasingly emphasizes cognitive processes in social contexts, with growing attention to socially shared retrieval-induced forgetting (SS-RIF). Behavioral evidence suggests that the mechanisms of SS-RIF are fundamentally aligned with those of retrieval-induced forgetting (RIF). However, SS-RIF differs from RIF due to the influence of social interaction factors. This study investigated the cognitive neural mechanisms of SS-RIF, focusing on its commonalities and distinctions from RIF. **Methods:** Experiment 1 examined neural evidence associated with retrieval-related processes. Twenty-eight university students participated in a within-subject design including retrieval practice, social retrieval practice, and baseline conditions. Correct recall rates and P2/FN400 amplitudes were measured. Experiment 2 investigated the temporal dynamics of inhibitory mechanisms by narrowing the design to SS-RIF only, using a within-participant design with first, second, and third repetition blocks occurring within the social retrieval phase, and recall was tested across two days. **Results:** Classic RIF and SS-RIF effects were observed, accompanied by neural activity indicative of inhibition during both retrieval practice and social retrieval conditions. These findings are consistent with the hypothesis that SS-RIF and RIF may share overlapping underlying cognitive operations. Typical RIF and SS-RIF phenomena were also observed across repetition conditions, while neural data showed no significant differences in P2 and FN400 amplitudes for SS-RIF, highlighting a distinction from patterns observed in RIF. **Conclusions:** Cognitive suppression accounts offer a compelling framework for explaining SS-RIF during social retrieval practice. However, compared with RIF, the duration of inhibition differs in SS-RIF. Unlike RIF, SS-RIF shows no reduction in specific EEG signal amplitudes over time. These findings provide cognitive neural evidence for inhibitory mechanisms in social retrieval and preliminary insights into the shared and distinct mechanisms of socially shared and individual RIF.

## 1. Introduction

In everyday life, people often discuss and recall shared experiences [1], a process termed collaborative recall [2,3]. During such conversations, individuals may selectively share specific details, either intentionally or unintentionally [4,5]. Research indicates that selective retrieval may lead speakers to forget relevant, unmentioned information, a phenomenon termed within-individual retrieval-induced forgetting (RIF) [6]. Moreover, selective retrieval by speakers can indirectly prompt listeners to forget relevant yet unmentioned information. Cuc et al. [3] introduced socially shared retrieval-induced forgetting (SS-RIF), a phenomenon where speakers’ retrieval of shared experiences leads listeners to forget related details.

In their study, Cuc et al. [3] had two participants independently learn all exemplar categories. During the social retrieval phase, one participant acted as the speaker, engaging in retrieval practice, while the other participant served as the listener. The speaker practiced retrieval across multiple categories using prompt cue–exemplar pairs. Conversely, the listener only listened to the speaker’s content and did not overtly participate in retrieval practice. During the final recall phase, participants recalled words learned in the study phase using category cues. Exemplars were categorized into three groups: practiced items (Rp+, representing successfully retrieved items associated with the category cue), unpracticed items from the same category (Rp−, representing non-retrieved items that share the same category), and unpracticed items from unrelated categories (Nrp, serving as the baseline). Within this design, the initial association strength of these items to their category cue is critically distinguished as high association (+) or low association (−) to balance competition dynamics. A significantly lower recall accuracy for Rp− exemplars compared to Nrp exemplars in speakers indicates the presence of RIF. If listeners exhibit lower recall rates for Rp− compared to Nrp, it implies that the speaker’s selective retrieval indirectly affects the listener’s memory, causing them to forget relevant information. This phenomenon is termed socially shared retrieval-induced forgetting (SS-RIF).

SS-RIF is hypothesized to result from covert retrieval by the listener. Research indicates that SS-RIF in listeners may involve mechanisms akin to those of RIF in speakers, with both analyzable within a shared theoretical framework [7,8]. Two primary theories explain individual-level RIF: inhibition theory and non-inhibition (or interference) theory [9]. Inhibition theory, the leading explanation for individual-level RIF [6,10], proposes that category cues activate both Rp+ and Rp− exemplars during retrieval practice. Individuals actively suppress the memory representations of exemplars to enhance Rp+ retrieval and minimize Rp− interference. Inhibition theory asserts that RIF arises only when retrieval practice is limited to the same category as the retrieval cue, free from interference by practiced items [6,11,12]. Interference theory offers an alternative explanation, proposing that retrieval practice reinforces the link between target exemplars and category cues. However, RIF occurs due to the disruption of connections between unpracticed exemplars and retrieval cues. This interference affects Rp− recall in the final test, irrespective of exemplar strength. Rp+ interferes with unpracticed Rp− exemplars, leading to lower recall rates than non-retrieved exemplars (Nrp) [13]. In social retrieval practice, Abel et al. [7] demonstrated that inhibitory mechanisms account for forgetting in both listeners and speakers, as retrieving target exemplars inhibits related exemplars. Participants completed four experiments involving diverse recall tasks. In Experiment 1, participants performed a retrieval practice paradigm, with listeners and speakers recalling categories in the final phase.

Experiment 2 used a recall cue by presenting the category word and its initial letter. In Experiments 3 and 4, participants identified words presented on a screen. Additional words were displayed, and participants indicated whether they had originally learned them. The study found that both listeners and speakers exhibited retrieval-induced forgetting across all recall tests, indicating that specific recall cues did not affect the SS-RIF effect in listeners. Therefore, the RIF phenomenon in both listeners and speakers likely stems from the same cognitive mechanism, primarily driven by inhibition.

Prior studies suggest that RIF and SS-RIF emphasize distinct aspects of memory inhibition [7]. RIF has been extensively studied from behavioral and cognitive neuroscience perspectives, whereas SS-RIF research has largely focused on behavioral evidence to establish its presence. Johansson et al. [14] performed the first ERP study on RIF, showing that retrieval practice increases positive ERP activity in frontal regions. Competitive conditions are associated with reduced FN400 amplitudes relative to non-competitive conditions, indicating heightened inhibitory processes in the frontal regions [15]. Studies indicate that repeated retrieval practice diminishes the amplitude of the frontal FN400 component [16]. The FN400 component, linked to the reactivation of competing memories, suggests that competition activates inhibitory mechanisms to reduce interference, supporting the role of inhibition in RIF [17]. Recent research has applied cognitive neuroscience techniques to examine SS-RIF.

Stephens et al. [18] employed fMRI to analyze brain activity during SS-RIF in both speakers and listeners. Similar brain activation patterns were observed in listeners when they heard recordings of speakers [18,19]. Retrieval in speakers activated the Default Mode Network (DMN), encompassing the posterior cingulate cortex and medial prefrontal cortex. The posterior cingulate cortex, central to higher cognitive functions, plays a key role in autobiographical memory retrieval within the DMN. The medial prefrontal cortex (MPFC) is a critical component of episodic memory systems in diverse contexts [20]. This evidence highlights that SS-RIF extends beyond behavioral phenomena to involve neural correlates.

This study first examines the behavioral outcomes and neural activity linked to SS-RIF, aiming to identify its similarities to RIF. Additionally, we investigated unique cognitive inhibition characteristics of SS-RIF. To address this, we designed Experiment 1, which involved three tasks: a retrieval practice task, a social retrieval practice task, and a relearning (baseline) task. The retrieval practice task included four phases: learning, consolidation, a filler task, and a final recall. This allows us to examine and compare the behavioral outcomes and neural activities across these conditions. Previous research indicates that inhibition occurs exclusively during retrieval, leading to forgetting of the target exemplar. Merely viewing or learning the exemplar does not activate inhibitory mechanisms or cause forgetting [14,21]. In other words, inhibition of Rp− items was primarily observed in the retrieval practice and social retrieval tasks, prompting us to compare neural activities across these two conditions. Additionally, comparing neural activity across the baseline, retrieval practice, and social retrieval practice conditions ensured the validity of the experimental procedure. This also confirmed that participants understood the task and that proper retrieval manipulation was conducted [22].

It is worth noting that within the context of retrieval-induced forgetting, the term ‘inhibition’ is used here strictly to denote cognitive suppression at the psychological and executive control level, rather than physiological neuronal inhibition at the cellular or synaptic level. Furthermore, we acknowledge that standard behavioral metrics (Rp− < NRp−) and scalp-recorded ERP components cannot definitively adjudicate between cognitive inhibition and association-based interference accounts, as both theoretical frameworks predict highly overlapping data patterns. Nevertheless, we adopt the cognitive suppression framework as our preferred theoretical interpretation, given its historical efficacy in explaining active executive control over competing memory traces during target retrieval.

Therefore, we propose the following hypothesis:

**Hypothesis** **1.** *In retrieval practice conditions, speakers will show RIF, as indicated by a significantly lower recall rate for Rp− exemplars compared to NRp−, and this phenomenon will not occur in the relearning condition. This will be accompanied by significantly higher mean P2 and FN400 amplitudes than the baseline. In social retrieval practice conditions, listeners will demonstrate SS-RIF, marked by similar patterns of lower recall for Rp− exemplars and elevated P2 and FN400 amplitudes.*

Current research on RIF suggests that retrieval inhibition mechanisms are transient, flexible, and adaptive in nature. Inhibiting competing items temporarily facilitates target item retrieval [23]. This is reflected behaviorally, with inhibition gradually diminishing over time. Chan et al. [24] found that recall tests conducted 20 min and 24 h after retrieval practice showed Rp− items were recalled at a higher rate than NRp− items after 24 h. Thus, Experiment 2 aims to explore the temporal characteristics of SS-RIF and identify its distinct features compared to RIF. In addition, previous RIF studies have demonstrated the existence of correlated patterns of neural activity [17,25]. Kuhl et al. [26] used fMRI to reveal that inhibition occurs during repetitive retrieval when competing memories conflict with the target memory. An EEG study found that theta activity, initially elevated during selective retrieval, decreased over time as competing representations were successfully suppressed [27]. Furthermore, retrieval practice inhibited theta waves more than relearning, resulting in a significant reduction in theta activity with repeated practice [25]. ERP studies have shown that repeated retrieval practice significantly reduces the frontal FN400 component [17]. These patterns indicate that neural activity is more pronounced in earlier repetition blocks and diminishes over time. This experiment compares neural activity across different retrieval blocks to investigate the temporal characteristics of SS-RIF’s inhibitory effects. Therefore, our second hypothesis is:

**Hypothesis** **2.** *The SS-RIF condition will demonstrate neural activity characterized by inhibition, akin to the RIF condition, with enhanced inhibition in the initial phases and reduced inhibition in later stages. Consequently, the mean amplitudes of the FN400 and P2 components will be observed to be higher in comparison to those recorded in the late block.*

## 2. Materials and Methods

### 2.1. Experiment 1

#### 2.1.1. Participants

Sample size calculation was performed using G*Power (ver. 3.1.9.6), indicating the need for 28 participants to detect a large effect size of at ηp2 = 0.25 with a standard statistical power (1 − β = 0.80; α = 0.05, two-tailed). Thirty-one adults (M = 19.96, SD = 1.79) were recruited for the study. Three participants whose final recall scores deviated more than three standard deviations from the mean were excluded from analysis. As a result, 28 participants remained in the study (M = 19.43, SD = 1.50). All participants were right-handed, had normal or corrected-to-normal vision, and reported no history of neurological or psychiatric disorders. Informed consent was obtained from all participants, who were informed that they would receive a fee for participation. The experiment was approved by the Institutional Review Board of the Academy of Psychology and Behavior, Tianjin Normal University, China (No. 2024012437).

#### 2.1.2. Task and Materials

In our experiment, a 3 × 4 within-subject design was used, with experimental condition (retrieval practice, social retrieval, and relearning) and item type (Rp+, Rp−, Nrp+, and Nrp−). The category–exemplar word pairs used in this study were sourced from the Chinese category–exemplar word list [28]. Twelve categories were selected as experimental materials. One additional category was selected as filler material. For each category, a set of 12 exemplar words was selected and divided into six high-association and six low-association items relative to the category [29]. During the retrieval practice phase, exemplar words weakly associated with the category and successfully retrieved were classified as Rp+ items; those belonging to the same category but not retrieved, with strong associations, were classified as Rp− items. To control for the influence of association strength, exemplar words in non-retrieved categories were divided into Nrp+ (low association) and Nrp− (high association) items [29]. A significant difference in word frequency was found between high-association and low-association items. Further details on the experimental materials can be found in the Supplementary Materials.

**Figure 1:**
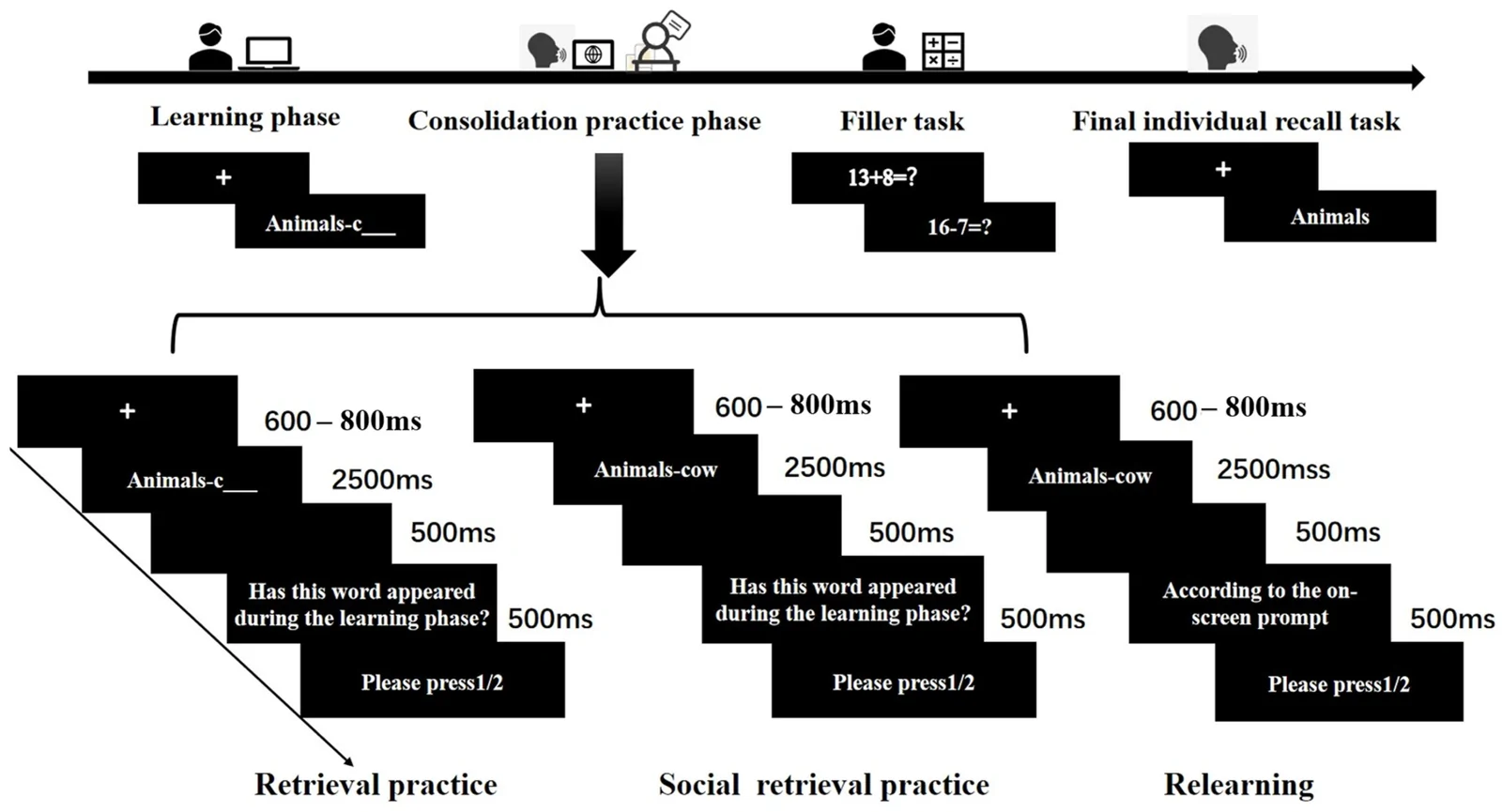
Procedures of Experiment 1. The experiment consisted of 4 phases. Of these, the consolidation-of-linkages phase includes three conditions (retrieval, social retrieval, and relearning).

Participants completed three experimental tasks, with conditions assigned in a pseudorandom order. Prior to the experiment, participants were informed they would complete three different tasks. Each task consisted of four phases: a learning phase, a consolidation practice phase, a filler task phase, and a final individual recall phase (see Figure 1). Participants were informed that EEG data would be recorded during the tasks. The entire session lasted approximately 100 min. These procedures are further detailed below.

Learning phase: Participants were instructed to memorize category–exemplar pairs (e.g., animals–mouse; animals–cow) presented in a pseudorandom order. Each pair appeared centrally on a screen for 5 s, with a 1.5 s interstimulus interval. Participants were instructed to memorize them for a later test. Filler pairs were presented at the beginning and end of the list during the learning and retrieval phases to control for primacy and recency effects [30]. The remaining exemplars were presented in a fixed order to avoid consecutive presentations of the same category.

Consolidation practice phase: In the retrieval practice phase, participants repeatedly retrieved half of the exemplars from category words using prompts (e.g., animals–tig_), referred to as Rp+ items. After retrieval, participants pressed keys to indicate whether the words had appeared during the learning phase (1 for yes; 2 for no). To minimize myoelectric artifacts in EEG recordings, participants were instructed to perform retrieval silently without verbal reporting [14].

In the social retrieval practice phase, the experimenter enlisted a confederate to assist participants in completing the task. Both the confederate and participants learned the same word pairs in the learning phase. The instructions for this phase were: “Another student will join you. During the retrieval phase, they will complete the task on another computer through the experimental platform. You will observe their input on the screen and complete the corresponding tasks.” In this phase, participants observed word pairs “completed” by the confederate (Rp+) and judged whether the words appeared in the study phase by clicking a button (1 for yes; 2 for no). There are 3 repeated blocks for each condition, and each word pair appears twice in each block.

In the relearning condition, participants observed and learned half of the exemplars from category words (Rp+) (see Johansson for a similar procedure) [14]. To control for irrelevant variables, participants were required to perform a key task (pressing 1 or 2 based on screen prompts) during the relearning condition, though accuracy judgment was not required. The experimental flow for the three conditions—retrieval practice, social retrieval practice, and relearning—is shown in Figure 1.

Filler task phase: A 5-min filler task was given between the consolidation practice phase and the final recall phase. During this period, participants completed a mathematical calculation task (e.g., 22 − 7 = ?).

Final individual recall phase: In this phase, participants recalled as many previously learned exemplar as possible based on category cues. Since no EEG data were recorded in this phase, participants recalled the words aloud, and the experimenter recorded the responses.

#### 2.1.3. Data Acquisition

E-prime 3.0 software (Psychology Software Tools, Inc., Pittsburgh, PA, USA) was used to present the stimuli and collect behavioral data. EEG data were acquired using a 64-channel amplifier and electrode cap, based on the extended International 10-20 EEG system. All electrode impedances were kept below 5 kΩ, and data were sampled at 500 Hz (DC). Vertical electro-oculograms (EOGs) were recorded above and below the right eye, while horizontal EOGs were recorded at the edges of both eye sockets. Data were processed offline using a superimposition method [31].

#### 2.1.4. Data Analysis

Behavioral data were analyzed using SPSS 26.0. A 3 × 4 repeated-measures ANOVA was performed on participants’ correct recall rates, with experimental conditions (retrieval practice, social retrieval practice, and relearning) and item types (Rp+, Rp−, Nrp+, and Nrp−) as factors. The significance level was set at *p* < 0.05, with Bonferroni correction applied for post hoc multiple comparisons.

EEG data were analyzed using the EEGLAB toolbox (Swartz Center for Computational Neurosciences, La Jolla, CA, USA; http://sccn.ucsd.edu/eeglab (accessed on 31 March 2025)) [32], involving the following steps: (1) channel localization; (2) re-referencing the data to bilateral mastoids (M1, M2); (3) removal of electro-oculogram (horizontal and vertical); (4) high-pass filtering from 0.01 Hz to 40 Hz; (5) ocular artifact correction using independent component analysis and visual inspection of trials; (6) segmentation of filtered data based on markers, spanning from −500 ms to 2000 ms; (7) baseline correction (500 ms before stimulus); (8) deartifacting, rejecting EEG signals exceeding ±80 μV; and (9) overlapping averages across conditions and participants. ERP analyses focused on neural activity during the consolidation phase, specifically examining P2 and FN400 components in response to word presentation, based on previous research. The P2 component (time window 210–270 ms) was analyzed at Fc1, Fcz, and Fc2 electrode sites [33]. The FN400 component (time window 410–500 ms) was analyzed at F3, Fz and F4 electrode sites [15]. Finally, the obtained data were statistically analyzed using SPSS 26.0. By standard electrophysiological convention, negative voltage values were plotted downwards, and variations in the FN400 component were characterized by changes in its negative deflection (where a reduction in memory conflict corresponds to a less negative or more positive deflection).

### 2.2. Experiment 2

#### 2.2.1. Participants

The sample size calculation was conducted using G*Power. A total of 29 participants were required to detect an effect size of ηp^2^ = 0.25 with a standard statistical power (1 − β = 0.80, α = 0.05, two-tailed). Twenty-eight adults (M = 19.00, SD = 1.00) were recruited for the experiment. All participants were right-handed, had normal or corrected-to-normal vision, and reported no history of neurological or psychiatric disorders. No participants were excluded based on the screening criteria established in Experiment 1. All participants provided written informed consent and received compensation for their participation. The study was approved by the Institutional Review Board of the Academy of Psychology and Behavior, Tianjin Normal University, Tianjin, China (No. 2024012437). Participants in Experiment 2 differed from those in Experiment 1 to prevent potential confounding and practice effects arising from the reuse of identical experimental materials.

#### 2.2.2. Task and Materials

Experiment 2 solely investigated the SS-RIF phenomenon, with item types (Rp+, Rp−, Nrp+, and Nrp−) as the independent variable in terms of behavior. In addition, different blocks (1, 2, and 3) were introduced at the neural level to verify whether the temporal characteristics of the inhibitory mechanism manifested in RIF will also appear in SS-RIF. The dependent variables were the participants’ recall rates for exemplar and their electrophysiological signals (P2 and FN400) across conditions. The materials used were the same as in Experiment 1.

Learning phase: Participants individually memorized category–exemplar pairs presented in a pseudorandom order.

Social retrieval practice phase: An experimental assistant acted as the speaker and collaborated with participants, following procedures outlined in Experiment 1. Unlike Experiment 1, Experiment 2 focused on changes in brain activity across blocks. This phase consisted of three repeated blocks, with each word being repeated twice within each block, totaling six repetitions.

Filler task phase: Participants performed simple subtraction tasks and responded via key presses to on-screen questions.

Final individual recall phase: In this phase, participants independently recalled as many exemplars as possible from each category studied.

The experiment spanned two days. Day 1 included four phases: learning, social retrieval, filler task, and final individual recall. On Day 2, participants recalled words learned during Day 1. The purpose of doing this is to verify whether the retrieval-induced forgetting effect will persist into the test on the following day (see also Valle et al.) [34].

#### 2.2.3. Data Acquisition and Analysis

Equipment and data collection procedures and behavioral data analysis were identical to Experiment 1. This experiment focused on changes in neural activity across repetition blocks. Experiment 2 analyzed the same EEG components (P2 and FN400). P2 (210–270 ms) was examined at the Fc1, Fcz, and Fc2 electrode sites [33]. FN400 (410–500 ms) was analyzed at the F3 and F4 electrode sites [15]. Data were statistically analyzed using SPSS 26.

## 3. Results

### 3.1. Experiment 1

#### 3.1.1. Percentage of Correct Responses in the Recall Phase

Table 1 presents participants’ recall rates for Rp and Nrp exemplars under three experimental conditions during the individual recall phase.

A repeated-measures ANOVA was conducted with three experimental conditions (retrieval practice, social retrieval practice, and relearning) and four item types (Rp+, Rp−, Nrp+, and Nrp−). For this part of the data, please refer to Supplementary Material Tables S1–S4. The results revealed a significant main effect of item type, *F* (3, 25) = 119.23, *p* < 0.001, η2 = 0.82. Post hoc comparisons showed that the recall rate for Rp+ exemplars (0.63 ± 0.16) was significantly higher than that for Nrp+ exemplars (0.32 ± 0.16), *p* < 0.001, 95%CI = [0.25, 0.39]. The recall rate for Rp− exemplars (0.22 ± 0.16) was significantly lower than that for Nrp− exemplars (0.37 ± 0.17), *p* < 0.001, 95%CI = [−0.21, −0.09].

The main effect of the experimental condition was not significant, *F* (2, 26) = 2.15, *p* = 0.126, η^2^ = 0.07. A significant interaction was observed between experimental conditions and item type, *F* (6, 22) = 10.34, *p* < 0.001, η^2^ = 0.28.

Simple effects analysis revealed that, under the retrieval practice condition, Rp+ exemplars were recalled significantly more often than Nrp+ exemplars, *p* < 0.001, 95%CI = [0.31, 0.53], while Rp− exemplars were recalled significantly less often than Nrp− exemplars, *p* < 0.001, 95%CI = [−0.39, −0.19]. These results confirm the classic phenomenon of retrieval-induced forgetting (RIF).

Under the social retrieval practice condition, Rp+ exemplars were recalled significantly more often than Nrp+ exemplars, *p* < 0.001, 95%CI = [0.25, 0.42], while Rp− exemplars were recalled significantly less often than NRp− exemplars, *p* < 0.001, 95%CI = [−0.25, −0.07]. These findings demonstrate the classic phenomenon of socially shared retrieval-induced forgetting (SS-RIF).

**Figure 2:**
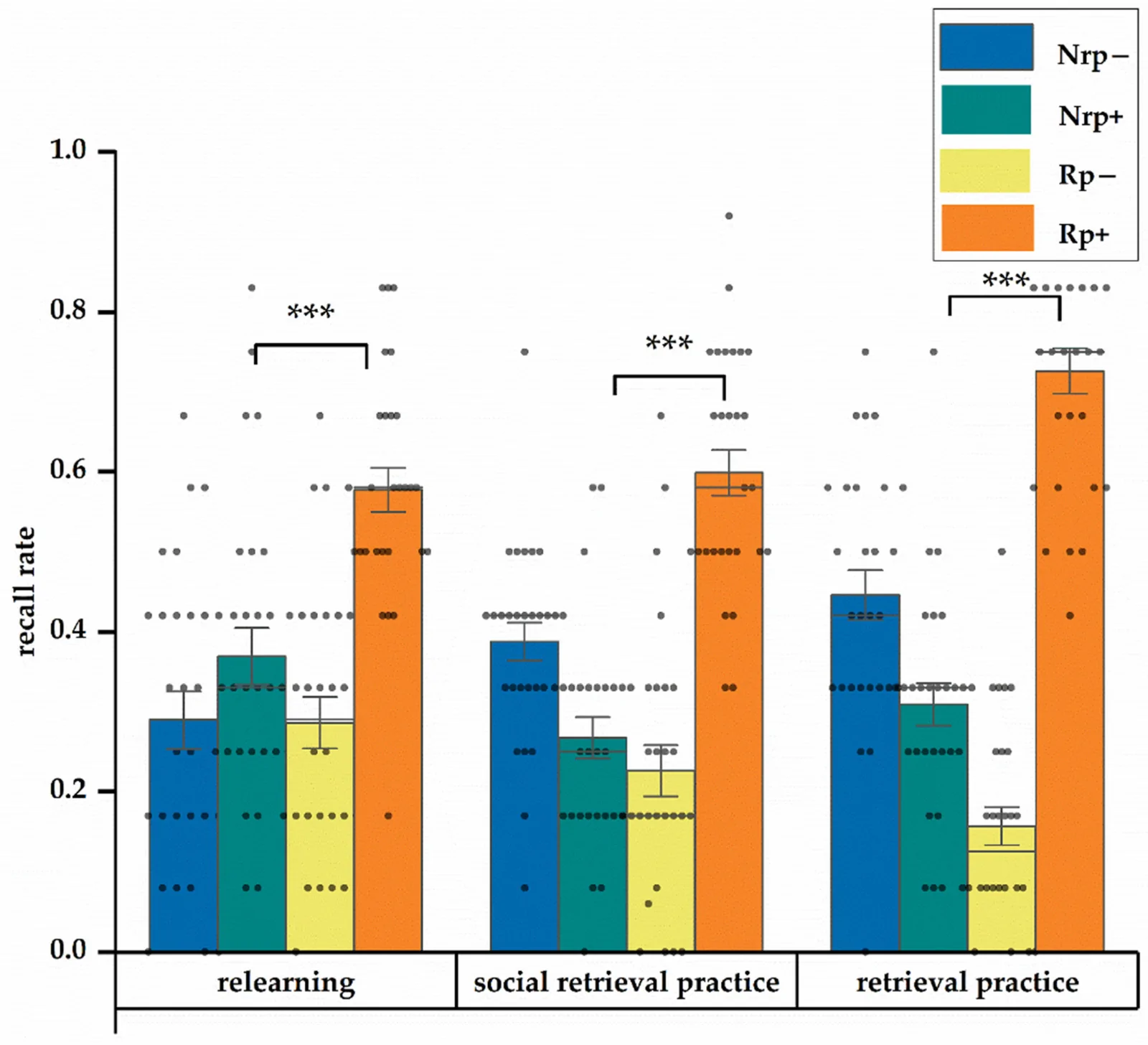
Recall rates for different item types. The *x*-axis represents conditions (retrieval practice, social retrieval, and relearning), and the y-axis indicates recall rates. The gray dots represent individual data points. The lines represent SE. Note: *** *p* < 0.001.

Under the relearning condition, Rp+ exemplars were recalled significantly more often than Nrp+ exemplars, *p* < 0.001, 95%CI = [0.10, 0.32]. There was no significant difference between the recall rates of Rp− and Nrp− exemplars, *p* = 0.470 These results suggest that repeated learning does not induce forgetting of Rp− exemplars (see Figure 2).

#### 3.1.2. ERP Results

Statistical analyses were conducted using SPSS 26.0. The P2 component (time window: 210–270 ms) was analyzed through a 3 (experimental conditions: retrieval practice, social retrieval practice, and relearning) × 3 (electrode sites: Fc1, Fcz, and Fc2) repeated-measures ANOVA.

P2 component: To account for potential violations of sphericity common in dense-array ERP data (see also [35,36,37]), a multivariate repeated-measures ANOVA (using Pillai’s trace criterion) was performed. The P2 amplitude showed no significant main effect of electrode sites, *F* (2, 26) = 2.38, *p* = 0.112, η^2^ = 0.16. The main effect of experimental conditions was significant *F* (2, 26) = 5.43, *p* = 0.011, η^2^ = 0.30. The interaction effect was not significant, *F* (4, 24) = 1.03, *p* = 0.411, η^2^ = 0.15.

**Figure 3:**
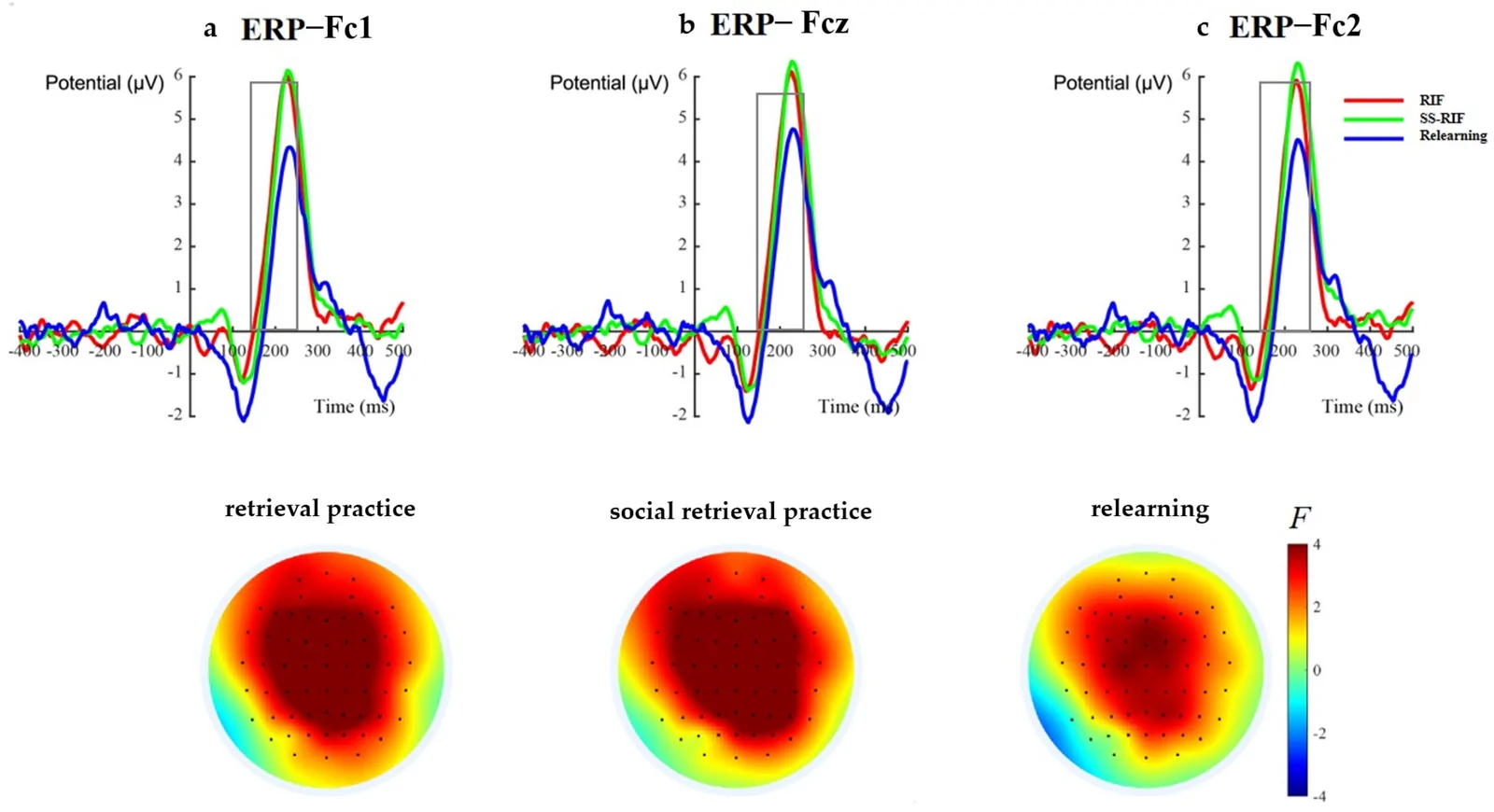
Average waveforms and topographic maps of P2 under three conditions. (**a**–**c**) Waveform diagrams for electrode sites Fc1, Fcz, and Fc2. Below each waveform, corresponding topographic maps for the three conditions are presented.

Since the interaction effect was not significant, post hoc comparisons were performed for the main effects. The Bonferroni correction post hoc comparison indicates no significant difference was observed between retrieval practice and relearning conditions, *p* = 0.158, 95%CI = [−0.53, 4.58]. A significant difference was found between social retrieval and relearning conditions, *p* = 0.007, 95%CI = [0.39, 2.92], and no significant difference between retrieval practice and social retrieval conditions (*p* = 1.000, 95%CI = [−1.78, 2.53]), as shown in Figure 3.

To further evaluate the null contrast between individual retrieval practice and social retrieval practice, supplementary paired-samples Bayesian t-tests were conducted on the P2 amplitudes. The analysis yielded substantial empirical evidence supporting statistical equivalence, revealing Bayes factors of *BF*_01_ =4.8 at Fc1, *BF*_01_ =4.56 at Fcz and *BF*_01_ = 4.16 at Fc2, confirming that the lack of difference across these sites is strongly supported by the empirical data.

FN400: Consistent with the P2 analysis, a multivariate repeated-measures ANOVA (using Pillai’s trace criterion) was applied to the FN400 amplitudes. The FN400 component (time window: 410–500 ms) was analyzed using a 3 (experimental conditions: retrieval practice, social retrieval practice, and relearning) × 3 (electrode sites: F3, Fz, and F4) repeated-measures ANOVA.

**Figure 4:**
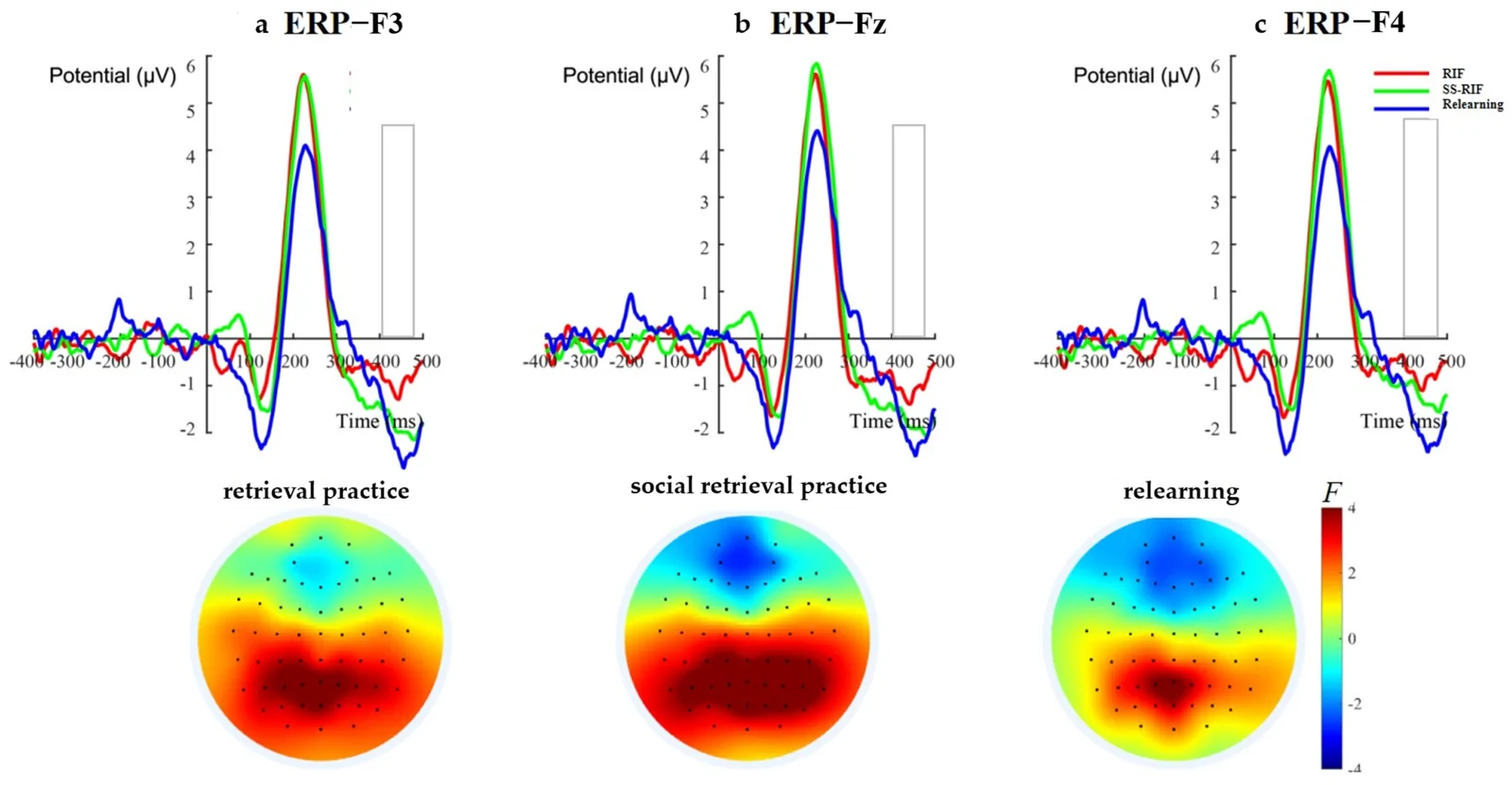
Average waveforms and topographic maps of FN400 under three conditions. (**a**–**c**) Waveform diagrams for electrode sites F3, Fz, and F4, with corresponding topographic maps shown below.

The main effect of experimental conditions was significant, *F* (2, 26) = 3.71, *p* = 0.038, η^2^ = 0.22. The main effect of electrode sites approached significance, *F* (2, 26) =2.62, *p* = 0.092, η^2^ = 0.17. The interaction effect was not significant, with *F* (4, 24) = 1.31, *p* = 0.293, η^2^ = 0.18. Post hoc tests showed a significant difference was found between retrieval practice and relearning conditions, *p* = 0.033, 95%CI = [0.09, 2.74], with the retrieval practice condition eliciting a significantly less negative FN400 deflection than the relearning condition. No significant difference was observed between social retrieval and relearning conditions (*p* = 1.000, 95%CI = [−1.58, 2.44]) and between retrieval practice and social retrieval (*p* = 0.619, 95%CI = [−0.96, 2.93]), as shown in Figure 4.

To further evaluate the null contrast between individual retrieval practice and social retrieval practice, supplementary paired-samples Bayesian t-tests were conducted on the FN400 amplitudes. The analysis consistently yielded empirical support for statistical equivalence, with Bayes factors of *BF*_01_ = 2.90 at F3, *BF*_01_ = 3.11 at Fz, and *BF*_01_ = 4.34 at F4.

#### 3.1.3. Discussion

Experiment 1 employed event-related potential (ERP) technology to examine the role of inhibitory mechanisms in RIF and SS-RIF. Behavioral results revealed a forgetting effect in both retrieval practice and social retrieval conditions, aligning with prior studies [3,6]. In contrast, no forgetting effect was observed in the relearning condition, supporting the hypothesis that repetitive learning does not induce forgetting of Rp− exemplars [14]. In this study, P2 amplitudes were higher under retrieval practice and social retrieval conditions compared to relearning, supporting the role of inhibitory mechanisms in socially shared retrieval-induced forgetting. EEG results indicated that RIF and SS-RIF exhibited significantly higher positive P2 amplitudes in the 210–270 ms time window compared to the relearning condition. This finding suggests that memory retrieval activities in the social retrieval condition resemble those in the retrieval practice condition, distinct from the relearning condition. Previous research has shown that retrieval inhibition involves enhanced positive brain activity in the early prefrontal cortex [14]. For the FN400 component (410–500 ms), the retrieval practice condition exhibited a less negative FN400 deflection compared to the relearning condition. The social retrieval condition also showed negative FN400 amplitudes, without significant differences from the relearning condition. However, a trend of a less negative deflection in the right frontal lobe was observed compared to the relearning condition. The FN400 component is linked to the reactivation of competing memories [34], suggesting that SS-RIF involves similar memory activation and inhibition processes as RIF. This finding partially reflects interference dependence during inhibition [38], ultimately leading to forgetting. These results suggest that during social retrieval, listeners engage in memory retrieval akin to speakers, leading to the activation and inhibition of unpracticed memories and subsequent forgetting. To further explore the duration and unique characteristics of socially shared retrieval-induced forgetting compared to retrieval-induced forgetting, the following experiment was conducted.

### 3.2. Experiment 2

#### 3.2.1. Percentage of Correct Responses in the Recall Phase

Table 2 presents participants’ recall rates for Rp and Nrp exemplars under three experimental conditions during the individual recall phase on Day 1 and Day 2.

To determine whether Day 1 findings replicated previous SS-RIF behavioral results, a repeated-measures ANOVA was performed on item types (Rp+, Rp−, Nrp+, and Nrp−). The results revealed a significant main effect of item type, *F* (3, 84) = 58.66, *p* < 0.001, η^2^ = 0.68. Post hoc tests indicated that the correct recall of Rp+ exemplars was significantly higher than that of Nrp+ exemplars, *p* < 0.001, 95%CI = [0.10, 0.19]. The recall rate of Rp− exemplars was significantly lower than that of Nrp− exemplars, *p* < 0.001, 95%CI = [−0.11, −0.05]. These results replicated previous findings on SS-RIF.

To examine whether SS-RIF persisted on Day 2, a repeated-measures ANOVA was conducted on recall rates by item type (Rp+, Rp−, Nrp+, and Nrp−). The results revealed a significant main effect of item type, *F* (3, 84) = 29.71, *p* < 0.001, η^2^ = 0.52. Post hoc comparisons showed that the recall rate for Rp+ items was significantly higher than that for Nrp+ items, *p* < 0.001, 95%CI = [0.08, 0.18]. No significant difference was observed between Rp− and Nrp− items, *p* = 0.752.

#### 3.2.2. ERP Results

Based on prior studies and average waveforms, six electrodes (Fc1, Fcz, Fc2, F3, Fz, and F4) were selected for statistical analysis [15,33]. ERP data were analyzed using SPSS 26.0 statistical software. Component amplitudes were analyzed in the P2 (210–270 ms) and FN400 (410–500 ms) time windows.

**Figure 5:**
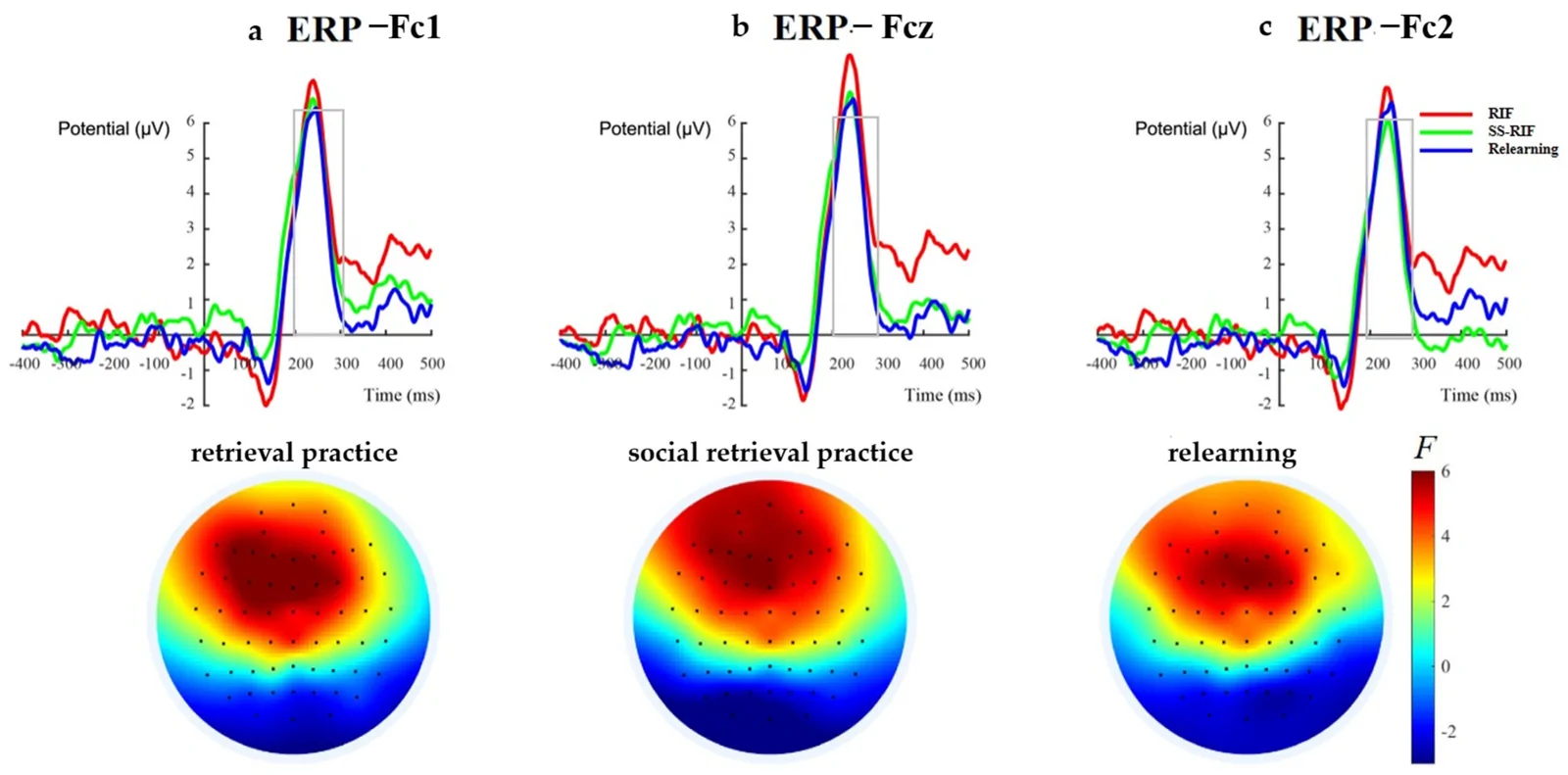
Average waveform plots for the P2 component across three blocks at three electrode sites. Subplots (**a**–**c**) show the waveforms for each electrode site. Below these, topographic maps illustrate the corresponding scalp distributions under three experimental conditions.

For P2 (210–270 ms), amplitudes were analyzed using a 3 (block: 1, 2, and 3) × 3 (electrodes: Fc1, Fcz, and Fc2) multivariate repeated-measures ANOVA (using Pillai’s trace criterion). The results revealed a significant main effect of electrodes, *F* (2, 27) = 6.91, *p* = 0.004, η^2^ = 0.34, but no significant main effect of block, *F* (2, 27) = 0.33, *p* = 0.722, η^2^ = 0.02, or their interaction, *F* (4, 25) = 1.33, *p* = 0.287, η^2^ = 0.18 (Figure 5).

To further validate the null main effect of blocks across time, supplementary paired-samples Bayesian *t*-tests were conducted on the P2 amplitudes. The analysis yielded substantial empirical evidence supporting statistical equivalence across all repeated blocks. Specifically, the contrasts against Block 3 revealed robust support for the null hypothesis, with Bayes factors of *BF*_01_ = 4.26 at FC1, *BF*_01_ = 3.34 at FCz, and *BF*_01_ = 4.96 at FC2 for the Block 1 vs. Block 3 contrast, and *BF*_01_ = 4.78 at FC1, *BF*_01_ = 5.04 at FCz, and *BF*_01_ = 4.25 at FC2 for the Block 2 vs. Block 3 contrast. Furthermore, the Block 1 vs. Block 2 contrast similarly favored the equivalence framework (*BF*_01_ = 4.46 at FC1, *BF*_01_ = 3.01 at FCz, and *BF*_01_ = 3.82 at FC2).

**Figure 6:**
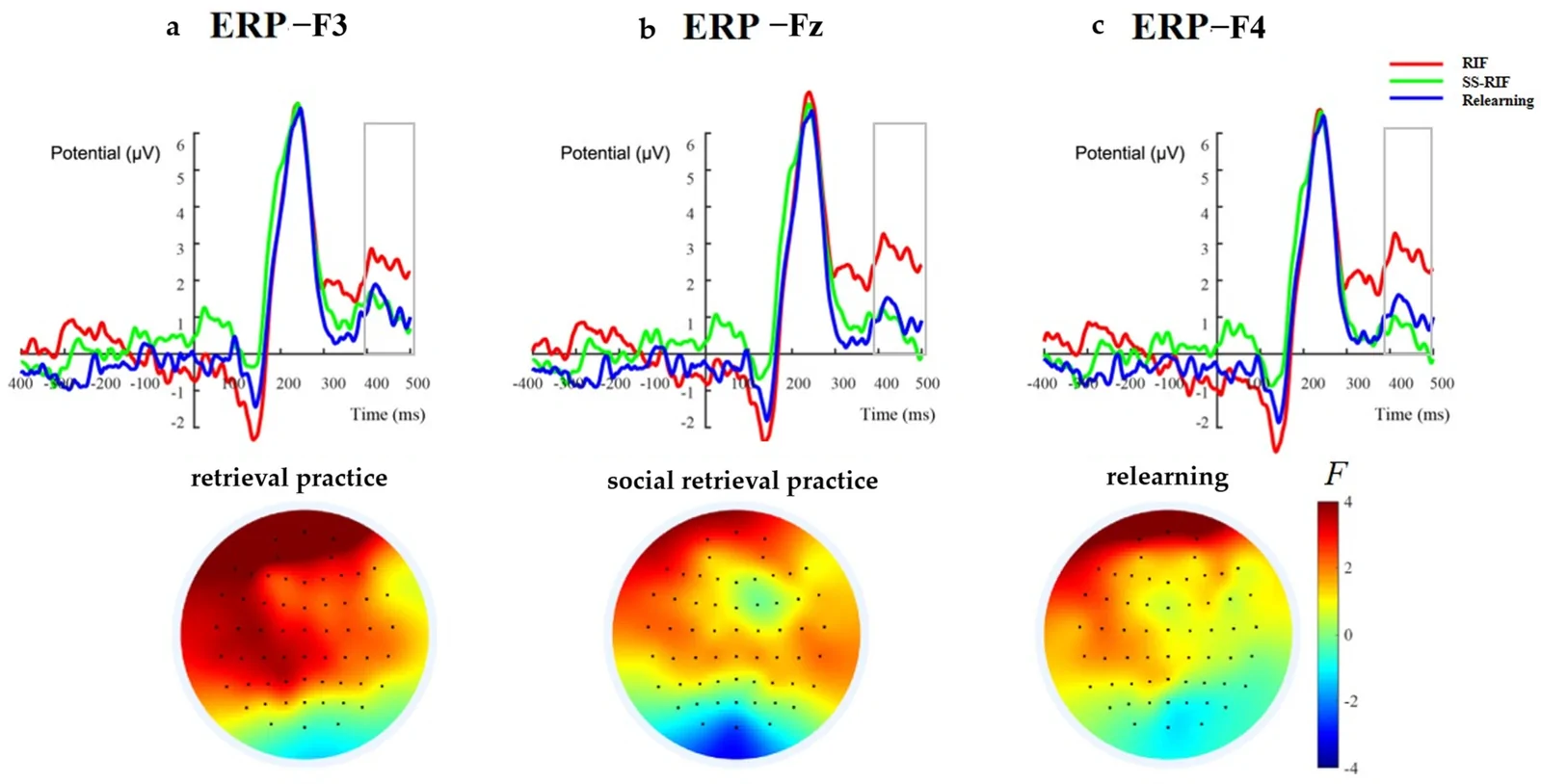
Average waveform plots for the FN400 component across three blocks at three electrode sites. Panels (**a**–**c**) depict the waveforms for each electrode site. Below these, topographic maps represent the scalp distributions under three experimental conditions.

Based on the overall waveform, FN400 amplitudes (410–500 ms) were analyzed using a 3 (block: 1, 2, and 3) × 3 (electrodes: F3, Fz, and F4) multivariate repeated-measures ANOVA (using Pillai’s trace criterion). The analysis revealed no significant main effect of electrodes, *F* (2, 27) = 0.13, *p* = 0.880, η^2^ = 0.01, or conditions, *F* (2, 27) = 1.13, *p* = 0.340, η^2^ = 0.08. The interaction effect was also not significant, *F* (4, 25) = 1.68, *p* = 0.187, η^2^ = 0.21 (Figure 6).

To address the null main effect of blocks with rigorous empirical baseline validation, supplementary paired-samples Bayesian t-tests were conducted on the FN400 amplitudes across different blocks. The analysis consistently yielded substantial evidence supporting the absence of block-wise differences (equivalence). Crucially, the statistical contrasts against Block 3 revealed robust support for the null hypothesis, with Bayes factors of *BF*_01_ = 4.31 at F1, *BF*_01_ = 3.82 at Fz, and *BF*_01_ = 4.04 at F2 for the Block 1 vs. Block 3 contrast, and *BF*_01_ = 5.05 at F1, *BF*_01_ = 4.91 at Fz, and *BF*_01_ = 4.74 at F2 for the Block 2 vs. Block 3 contrast. These Bayesian outcomes confirm that the observed stable temporal trajectory of the FN400 deflection across social interaction blocks is firmly supported by the empirical data.

#### 3.2.3. Discussion

Experiment 2 utilized event-related potential (ERP) technology to examine the duration characteristics of inhibition in socially shared retrieval-induced forgetting (SS-RIF). Behavioral results confirmed the classic phenomenon of SS-RIF. However, EEG results revealed no significant differences in P2 and FN400 components across blocks. This finding contrasts with the inhibitory mechanism characteristics observed in retrieval-induced forgetting [25,39]. In sum, these findings suggest that the inhibitory mechanism in SS-RIF is weaker compared to retrieval-induced forgetting.

## 4. Discussion

### 4.1. General Discussion

In this study, ERP technology was employed to compare the brain neural signals of participants under conditions of retrieval practice, social retrieval practice and relearning. The results revealed that both individual retrieval practice (RIF) and social retrieval practice (SS-RIF) conditions elicited distinct ERP modulations compared to the passive relearning condition. Specifically, the active practice conditions exhibited enhanced general retrieval engagement, as reflected by the modulation of the P2 and FN400 components. Among these three conditions, individual retrieval practice demonstrated the most pronounced ERP variations, followed by social retrieval practice, whereas passive relearning remained at the baseline. This pattern aligns with previous behavioral and theoretical accounts suggesting that active retrieval processes, and by extension the cognitive demands required for potential competition resolution, are recruited more heavily in individual retrieval-induced forgetting (RIF) than in SS-RIF [7]. By observing the EEG topographic maps, we found that the frontal scalp regions exhibited stronger positive or less negative deflections under the social retrieval practice condition. Although standard scalp ERPs cannot provide definitive anatomical localization due to intrinsic spatial-resolution limitations, this prominent frontal distribution is consistent with potential cognitive control networks that influence executive suppression. This suggests that the cognitive processes involved in social retrieval practice align with the engagement of inhibitory control and are involved in socially shared retrieval-induced forgetting, thereby providing robust evidence for interpreting these social memory phenomena through the lens of executive control models. In conclusion, this study replicated previous behavioral findings on socially shared retrieval-induced forgetting. It also identified similar inhibitory neural activities in both socially shared retrieval-induced forgetting and retrieval-induced forgetting (RIF). Additionally, differences in duration between SS-RIF and RIF were observed.

### 4.2. Behavioral Characteristics of Socially Shared Retrieval-Induced Forgetting

In Experiment 1, three conditions were manipulated during the consolidation phase: retrieval practice, social interaction retrieval, and relearning. Participants performed different tasks under each condition: In the retrieval practice condition, they completed the word completion task alone. In the social interaction retrieval condition, participants completed tasks based on the speaker’s performance. In the relearning condition, participants only reviewed and studied the learned word pairs.

Consistent with Anderson et al. [40], the baseline condition showed no significant difference in recall rates between Rp− and Nrp− exemplars, indicating no forgetting. This finding supports the view that forgetting due to specific inhibition occurs only when there is retrieval competition among relevant memory materials. In contrast, repeated viewing of items without competition leads to no forgetting [40].

In the retrieval practice conditions, participants had significantly lower recall rates for Rp− exemplars compared to Nrp− exemplars. Conversely, Rp+ exemplars were recalled at significantly higher rates than Nrp+ exemplars, demonstrating the retrieval facilitation effect [41,42] and retrieval inhibition effect [40]. In the social interactive retrieval condition, participants recalled Rp− exemplars significantly less than Nrp− exemplars, indicating the emergence of socially shared retrieval-induced forgetting [43]. It is worth noting that due to the characteristics of EEG technology and issues with auditory stimuli, typing was used as an alternative to the traditional “speaking” and “listening” modalities. Despite this change, SS-RIF still occurred. Similar manipulations have been reported in previous studies [44]. This emphasizes that SS-RIF is not solely determined by the mode of information reception but rather depends on covert interactions within social contexts, which trigger shared retrieval processes between the “listener” and the “speaker”. Therefore, specific characteristics inherent in social interactions, rather than variations in communication forms, are the key factors influencing SS-RIF. For example, previous research has found that socially shared retrieval-induced forgetting is only induced when real others are present, whereas presenting participants with audio materials and asking them to complete the same task does not induce socially shared retrieval-induced forgetting [30]. In this study, the two participants were also placed in the same room to create an authentic interactive setting, and the behavioral results ultimately confirmed the effectiveness of this approach. These findings further demonstrate that social attributes are crucial to SS-RIF, marking a significant difference from individual RIF.

In Experiment 2, during the social interaction retrieval phase, there were three repeated blocks, with each word being repeated twice in each block. On the second day of recall, there was no significant difference between Rp− and Nrp− items, which is consistent with previous findings in retrieval-induced forgetting [24]. This suggests that the suppression of Rp− exemplars did not persist after a day under the current paradigm. This finding aligns with the phenomenon of RIF [34]. This also show that, compared to RIF, the role of inhibitory mechanisms in SS-RIF is less pronounced, validating previous conclusions [7]. Furthermore, the behavioral results on the first day indicated the effectiveness of the manipulation, which induced the phenomenon of SS-RIF. On the second day, when the recall rates of Rp− and Nrp items were compared, it was found that the recall rate of Nrp items was still slightly higher than that of Rp− items. This result is different from that of previous RIF studies. In previous research [24], the recall rate of Rp− exemplars on the second day was higher than that of Nrp exemplars. Chan [24] suggested that the enhancing effect of retrieval practice on recall might last longer than the decrease in recall caused by retrieval-induced suppression. However, in this study, such a difference was not observed, which may be because suppression lasts longer in SS-RIF compared to RIF. In subsequent experiments, the testing time could be extended, drawing on the specific operations of RIF, from intervals of 24 h to three days, or even a week, to more meticulously study the long-term effects of suppression on Rp− exemplars over time.

### 4.3. Neural Mechanisms of Socially Shared Retrieval-Induced Forgetting

In Experiment 1, we recorded electroencephalogram (EEG) signals during the consolidation phase under three conditions: retrieval practice, social retrieval practice and relearning. P2 and FN400 components were selected as the primary signals for observation. In the field of active forgetting, the P2 component is associated with memory retrieval activities [33,45,46] and may reflect retrieval inhibition [14]. FN400 is associated with the reactivation of competing memories [15]. Firstly, comparing the P2 component, we found that both RIF and SS-RIF elicited more intense neural activity compared to the relearning condition. This indicates that participants showed memory-retrieval-related activities in both RIF and SS-RIF. This result aligns with previous research for the retrieval practice condition [14]. Prior studies suggest that “implicit retrieval” by listeners is key to socially shared retrieval-induced forgetting [9,47]. That is to say, although the listener did not directly complete the word completion task, they internally and silently extracted the content of the speech they heard while listening to the speaker performing the task. In this study, P2 neural activity under the social interaction retrieval condition, compared to the relearning, reflects this “silent” retrieval activity not observable through behavioral studies.

The P2 activity also reflects the inhibitory mechanism underlying SS-RIF. Johansson compared ERP neural activities across 24 participants under different conditions (retrieval practice, and relearning) [14]. Their findings showed that ERPs capture the temporal dynamics of inhibitory processes, notably with more positive ERP components in the prefrontal region during the early time window under retrieval practice. This study found that SS-RIF also elicited more positive ERP components in the early prefrontal cortex compared to the relearning condition. Since the prefrontal cortex is linked to cognitive control, including inhibitory control, working memory, and cognitive flexibility [48], this finding supports the role of inhibitory mechanisms in socially shared retrieval-induced forgetting.

Secondly, compared to the relearning condition, both the retrieval practice phase and the social interaction retrieval phase induce a less negative FN400 deflection. In RIF research, FN400 is related to the reactivation of competitive memories [15]. The less negative FN400 deflection indicates that the relevant memories also have a tendency to be activated, and this competition will trigger inhibitory mechanisms to suppress them, thereby allowing the target items to be activated smoothly. The FN400 component in retrieval-induced forgetting was significantly higher than the relearning, consistent with Valle et al. [34]. The FN400 observed in SS-RIF did not significantly exceed the relearning, but showed a trend above the baseline, which also indicates the presence of inhibition in the phenomenon of socially shared retrieval-induced forgetting. However, as acknowledged in our limitations, the behavioral manipulation of the social interaction condition structurally overlaps with the baseline condition to a certain extent. This resemblance requires a qualified and cautious interpretation of the subtle neural variations observed between these two conditions. When comparing both the P2 component and the FN400 component, we found no significant differences between the retrieval practice phase and the social interaction retrieval phase. Although a non-significant *p*-value in traditional hypothesis testing cannot definitively prove absolute equivalence, our supplementary Bayesian analysis for the P2 window (*BF*_01_ = 4.93) provides substantial statistical support for the absence of a difference. This also suggests that the two share some similar cognitive mechanisms, addressing the first question we wanted to explore in this paper: identifying some cognitive commonalities between retrieval-induced forgetting and socially shared retrieval-induced forgetting. Previous research suggested that RIF and SS-RIF have similar internal mechanisms. However, they are not entirely the same processes. The latter arises in social interactions [43] and is influenced by many factors of social interaction (such as gender, intimate relationships, etc.). Therefore, investigating the neural activity characteristics of SS-RIF is essential. In RIF, cognitive neural processes show intense activity in early repetition blocks and weaker activity in later blocks, with inhibition diminishing over time until it disappeared. Prior research indicates that the inhibitory effect in SSRIF is weaker than in RIF [7]. The results of this study support the views of previous researchers and indicate that the impact of SS-RIF is more enduring.

In Experiment 2, we recorded EEG signals during the social interaction retrieval phase and focused on P2 and FN400 as primary signals. Unlike Experiment 1, Experiment 2 subdivided the time course and compared P2 and FN400 neural activity across different repetition blocks. The results showed differences in neural activity intensity between repetition blocks, but these did not reach significance, differing from RIF. In previous research on the duration aspect, it has been found that there are significant differences in brain neural activity between different numbers of retrievals. In this study, when analyzing the brain signals during the social interaction retrieval phase, similar results were not found. However, by observing the waveforms in Experiment 2 of this study, it was found that brain activity was more intense in the first repetition block. This suggests that inhibitory activity persisted throughout, with waveform differences observed between repetition blocks, but the degree of inhibition did not show a significant trend of initially stronger and then weaker. This may be due to the weaker inhibitory effect of SS-RIF compared to RIF. Hence, differences between repetition blocks did not achieve significance. It is worth noting that the observed effect sizes across blocks were relatively small, meaning the present study might have limited statistical power to detect such subtle temporal fluctuations. Therefore, the non-significant block effects should be interpreted with caution, and future studies with larger sample sizes are required to further verify these temporal dynamics. Although this study has identified some cognitive and neural characteristics of SS-RIF through ERP experiments, there are still many technical limitations that deserve further breakthroughs. For instance, the manipulation of the social interaction retrieval condition in this experiment needs further refinement, as it overlaps and is similar to the baseline condition in many aspects. Therefore, the behavioral and brain results did not fully validate the hypotheses. Future studies should enhance the “social” attributes in the SS-RIF paradigm to further test the hypotheses. Additionally, in ERP experiments, data are often accumulated by repeating many blocks, which, while scientifically and completely facilitating our data, also increases the cognitive load on participants, making them feel fatigued. In a two-person experiment, this fatigue might be contagious, reducing the overall experimental ecological effect. Furthermore, the spatial resolution of ERP experiments is not high, preventing precise localization to deep brain areas, which means we cannot see the neural structural dynamics behind specific phenomena. In the future, a combination of EEG and MEG methods could be employed for a more comprehensive study of this phenomenon. It is also possible to combine EEG with eye trackers, polygraphs, and other tools to study SS-RIF from multiple dimensions and discover its various characteristics.

Additionally, future paradigms with a substantially higher number of trials per condition should implement a direct, within-study block-by-block analysis to directly compare the localized temporal decay trajectories of individual RIF versus SS-RIF within the same sample.

## 5. Conclusions

This study identified cognitive activity characteristics that align with the predictions of the inhibition framework in socially shared retrieval-induced forgetting (SS-RIF), mirroring the patterns typically observed in individual RIF. At the same time, cognitive activity characteristics that are different between SS-RIF and RIF were also identified. Comparative analysis of P2 and FN400 components showed no significant differences between retrieval practice and social interaction phases. Notably, SS-RIF shows distinct cognitive activity features influenced by social interaction factors, unlike RIF. Although SS-RIF inhibition is weaker than RIF, its effects may persist longer.

## Figures and Tables

**Table 1:** Correct recall rate for different exemplar types (*M* ± *SD*).

	Retrieval Practice	Social Retrieval Practice	Relearning (Baseline)
Rp+	0.72 ± 0.15	0.60 ± 0.15	0.58 ± 0.15
Rp−	0.16 ± 0.14	0.23 ± 0.17	0.29 ± 0.17
Nrp+	0.31 ± 0.14	0.27 ± 0.14	0.37 ± 0.19
Nrp−	0.44 ± 0.16	0.39 ± 0.12	0.29 ± 0.19

**Table 2:** Correct recall rate for two days of different item types (*M* ± *SD*).

	Day 1	Day 2
RP+	0.50 ± 0.11	0.47 ± 0.11
RP−	0.31 ± 0.08	0.31 ± 0.08
Nrp+	0.36 ± 0.11	0.34 ± 0.11
Nrp−	0.44 ± 0.16	0.34 ± 0.10

## Data Availability

The data supporting this study’s findings are available on request with the consent of the corresponding author. However, the data are not publicly available because they contain information that could compromise the privacy of the research participants.

## Supplementary Materials

The following supporting information can be downloaded at: https://www.mdpi.com/article/10.3390/brainsci16080814/s1, Table S1: Experiment 1_Behavioral_Data; Table S2: Experiment 1_Brain_Data; Table S3: Experiment 2_Behavioral_Data; Table S4: Experiment 2 Brain_Data.
